# A conceptual framework of urban food security and nutrition in low‐ and middle‐income country settings applied to the Asia‐Pacific region

**DOI:** 10.1111/mcn.13560

**Published:** 2023-09-14

**Authors:** Paula Griffiths, Emily Rousham, Sophie Goudet, Jessica Blankenship, Zivai Murira, Britta Schumacher, Emma Haycraft

**Affiliations:** ^1^ School of Sport, Exercise and Health Sciences Loughborough University Loughborough UK; ^2^ University of the Witwatersrand Johannesburg South Africa; ^3^ Dikoda, Nutrition Research London UK; ^4^ UNICEF, EAPRO Bangkok Thailand; ^5^ UNICEF Regional Office for South Asia, Leknath Marg Kathmandu Nepal; ^6^ World Food Programme Asia Pacific Headquarters Bangkok Thailand

## Abstract

A conceptual framework is presented for enhancing food security and nutrition in urban areas in low‐ and middle‐income countries, highlighting key influencing factors, including food supply chains, community food environments, community infrastructure and services, and numerous underlying individual and household determinants, such as behaviours and dietary practices.

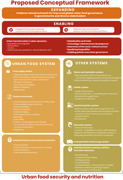

## 1 INTRODUCTION

Countries in the Asia‐Pacific region are experiencing rapid urbanisation (United Nations Economic and Social Commission for Asia and the Pacific (UNESCAP), 2019), which has complex and disparate effects on food security and nutrition among urban populations, including improvements for some urban residents and increased vulnerability for others. Slum living conditions, rising costs of food, decreasing purchasing power, unhealthy food environments and the expected impact of climate change are threats to food security and nutrition in cities (e.g., Tacoli, 2019). The increased prevalence of overweight and obesity alongside persistent underweight, stunting and micronutrient deficiencies resulting from these threats marks the triple burden of malnutrition in transition contexts (Congdon, 2019; Vilar‐Compte et al., 2021; World Health Organisation Regional Office for the Western Pacific, 2017). Responding to these challenges and protecting the most vulnerable groups, especially women and young children, in urban areas requires innovative policies and committed governance.

This special issue was born out of analyses undertaken on food security and nutrition in urban areas in the Asia‐Pacific region as part of the Asia and the Pacific Regional Overview of Food Security and Nutrition 2022 (FAO, UNICEF, WFP and WHO, 2023). The regional overview outlines why there is a need to focus on food security and nutrition in urban areas, how urban and rural areas differ, the food security and nutrition situation in the Asia‐Pacific region (including the impacts of COVID‐19), and the role of system‐level factors and determinants in food security and nutrition, and highlights innovations and opportunities to improve urban food security and nutrition.

## 2 A CONCEPTUAL FRAMEWORK OF THE DETERMINANTS OF URBAN FOOD SECURITY AND NUTRITION

Urbanisation has been increasing rapidly over the past few decades, and the population in Asia and the Pacific is now approaching 50% urban (United Nations Economic and Social Commission for Asia and the Pacific (UNESCAP), 2019). Given the scope and pace of growth of urban areas, achieving food security and adequate nutrition for the people living in urban areas will only be possible by building a sustainable food system and effectively integrating it with other systems, including water and sanitation, health, education, transport, energy, and social protection systems.

The conceptual framework presented within the Asia and the Pacific Regional Overview of Food Security and Nutrition 2022 can be linked to the Food and Agriculture Organization of the United Nations' (FAO) Urban Food Agenda as it provides a framework for enhancing food security, nutrition and sustainable development in urban areas. The FAO's approach to this agenda is based on the ‘3Es’ (enabling, executing and expanding) and the ‘comprehensive areas of support’ that align with the proposed framework's focus on enabling policy environments, executing context‐specific actions and expanding good practices.

The proposed conceptual framework for enhancing food security and nutrition in urban areas is particularly relevant because urban food security and nutrition are influenced by a range of factors. These factors include food supply chains, community food environments, community infrastructure and services, and a range of underlying individual and household determinants, such as behaviours and dietary practices.

**Figure 1:**
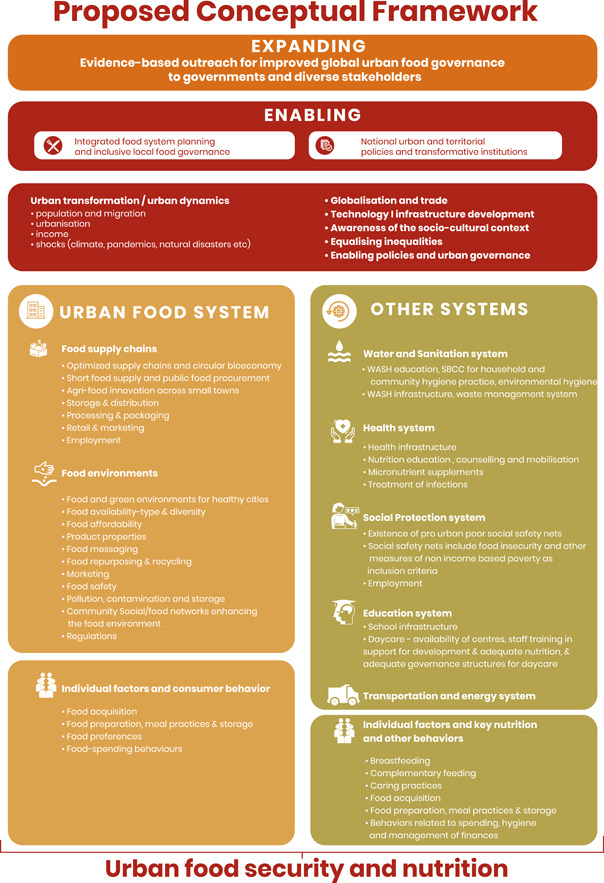
A conceptual framework showing determinants of urban food security and nutrition.

The conceptual framework (Figure 1) underlying the Asia and the Pacific Regional Overview of Food Security and Nutrition 2022 depicts the determinants of urban food security and nutrition that are considered by papers published as part of this special issue. Haycraft et al. (2023) consider the evidence base available to understand these relationships, Auma et al. (2023) look at individual factors and the relationship of these to nutrition and other behaviours, and Rousham et al. (2023) further our understanding of urban food environments. Both the papers and the Regional Overview report reflect recent impacts of the COVID‐19 pandemic on nutrition and food security.

The framework is needed because urban food systems in low‐ and middle‐income countries (LMICs) face some unique challenges compared to rural areas and larger shares of the population are living in urban areas in LMICs (e.g., United Nations Economic and Social Commission for Asia and the Pacific (UNESCAP), 2019). Urban food systems are impacted by numerous external drivers, including rapid urbanisation, structural transformation of the economy, climate change and the recent COVID‐19 pandemic. Urbanisation also drives economic growth and employment, which, in turn, increase food demand. Urbanisation puts pressure on the natural resources that food systems rely on and exacerbates vulnerabilities from climate change. This is particularly relevant in Asia and the Pacific, where countries are increasingly impacted by climate change (Intergovernmental Panel on Climate Change, 2022). These regions are home to some of the most vulnerable populations in the world, who are disproportionately affected by the consequences of climate change, such as extreme weather events, sea‐level rise and food insecurity. However, cities across LMICs are also able to provide income opportunities, which can ease household poverty and improve access to food.

Vilar‐Compte et al. (2021) previously developed a framework for nutrition and urban poverty based on a thematic analysis of systematic review evidence. The conceptual framework presented within the Asia and the Pacific Regional Overview of Food Security and Nutrition 2022 has a more ecological focus than the framework developed by Vilar‐Compte and colleagues. It adds enabling and expanding factors, factors associated with urban transformation and dynamics (e.g., migration and shocks), alignment with other important systems beyond food systems (e.g., water and sanitation, health, and social protection), as well as including aspects of urban transformation and dynamics often experienced in urban areas. However, there are fewer details on individual‐level factors in the conceptual model presented here than there are in the Vilar‐Compte et al. (2021) framework, with opportunities to learn from both models to guide thinking on food systems in poor urban areas at the different levels.

In this special issue, the papers consider in greater detail the different elements of the framework and provide evidence for the need to focus on nutrition and food systems for the urban poor.

Haycraft and co‐authors (2023) reveal a lack of data for the urban poor on which to base policy and food decision‐making systems. Only 13 countries in the Asia‐Pacific region had data since 2015 on maternal infant and young child nutrition (MIYCN) indicators, and, of these, only three had data on maternal indicators. None of the available population‐based survey data sets had information on children over the age of 5 years, men or older adults. Most of these countries also lack data from the poorest urban populations to understand MIYCN indicators for the urban poor. The data that do exist consistently reveal across all MIYCN indicators (other than immediate breastfeeding after birth) that urban slum residents fare worse than their non‐slum urban counterparts and that the poorest residents of slums have the poorest MIYCN outcomes. The paper identifies the need for better planning in population‐based national surveys such as Demographic and Health Surveys and Multiple Indicator Cluster Surveys to include the urban poor and slum communities within sampling frames. Many of these types of surveys still utilise census sampling frames which very quickly become outdated and tend to underrepresent informal settlements and new communities in urban settlements. Informal settlements are also the most likely to house the urban poor, who are particularly vulnerable to food insecurity, inadequate nutrition and threats from climate change. The paper advocates for more innovative ways of sampling, including the use of modern technologies such as aerial photography of urban spaces. When communities are not captured by maps and sampling frames, then food systems will also be less likely to adequately serve these areas.

Auma et al. (2023) build on our learning from the Haycraft et al. (2023) paper regarding the poorer outcomes for those living in slums to understand how nutrition indicators and food systems were further challenged during a period of shock (the COVID‐19 pandemic) in three Asia‐Pacific countries. Using case studies of Yangon in Myanmar, Jakarta in Indonesia and Quezon City in the Philippines based on Status and Determinants of Food Insecurity and Undernutrition Surveys (data collected remotely in 2020–2021) and comparing these to the most relevant available survey data before the COVID‐19 pandemic, the paper reveals that already vulnerable populations experienced reduced income. The paper also reports food systems disruptions that unequally affected the urban poor because they had fewer resources to fall back on in a crisis than other urban populations and were less likely to be producing their own food than their rural counterparts. As predicted (Pérez‐Escamilla et al., 2020), these factors resulted in larger numbers of households worrying about food compared to pre‐pandemic, and reductions in women and the youngest children being able to achieve minimum dietary diversity (MDD) during the pandemic. Nutrient‐dense foods were commonly substituted with less nutritious foods in all three cities. However, some positive messages emerge from the paper. In Yangon, households that received COVID‐19 relief payments were 43% less likely than households that did not receive such relief to consume no fruits and vegetables. In Jakarta, women were four times more likely to achieve MDD if the household received food subsidies, and in Quezon City, women were less likely to eat unhealthy food if the household received a food subsidy. This shows that cash assistance can have positive impacts on the diets of the poorest during a crisis. This finding is endorsed by Rousham et al. (2023), who highlight potential ways to support informal food vendors during future shocks like pandemics, including cash or voucher schemes for food, food safety and hygiene measures appropriate to the pandemic's risks, supporting smaller retailers with online skills, increasing links with smaller retailers and a registration system for local food vendors.

Rousham et al. (2023) reveal the impacts of modernising urban centres on the risks and opportunities for healthy diets for low‐income populations and consider the vulnerability of food systems in the Asia‐Pacific region using a case study example from the COVID‐19 pandemic. The authors highlight the need in urban centres to understand not only modern retail food outlets but also how these interact with wet markets and informal outlets, such as street food sellers, to provide nutritious and safe foods. Wet markets are a prominent feature of urban food environments in the Asia‐Pacific region and street foods provide an important opportunity for employment, especially for women, as well as access to food in areas that can be under‐served by other food outlets. The concerns around street foods often relate to hygiene problems, microbial contamination and weak context‐specific food safety regulation. The paper reveals that the Asia‐Pacific region experienced a 55% increase in the number of supermarkets between 2012 and 2017, greater than any other global region, but with marked variation in access to supermarkets across the region varying from 4.7% in Bhutan to 90% in South Korea and Malaysia. Affordability can also be a major barrier to using supermarkets or modern retail outlets for the urban poor. The paper also identifies the challenges of defining ‘food deserts’ (see Rousham et al. (2023) for definition of a food desert) compared to high‐income settings because often the population and food vendors are mobile, households might be producing their own food, and there are high risks of social, political and environmental shocks that rapidly change the situation. The paper shows the COVID‐19 pandemic shocks impacted already fragile food systems in the Asia‐Pacific region with limited options for food purchasing because food vendors closed down, there was less labour in agricultural centres and the longer food chains in urban centres made populations more vulnerable to food insecurity. This pushed up food prices alongside declining incomes, worsening food insecurity. Informal workers were most affected as they were often not eligible for social security protection systems. Some city governments intervened and purchased food and distributed this to the most vulnerable urban populations. Informal vendors were also the least likely to be able to switch to online trading, thus facing the worst income shocks. Rousham et al. (2023) conclude that enhancing urban food production will reduce transport, packaging and waste, but acknowledge that the challenges to this are high population density and demands on land, meaning there has to be high productivity per unit of land for it to be successful. They also identify the need to link urban farmers with modern retailers to fully realise these benefits.

Together the papers in this special issue highlight inequalities in urban areas relating to food security and nutrition. Understanding these urban inequalities is key to tackling the drivers of food insecurity and malnutrition in vulnerable urban communities. The Asia and Pacific region leads in technology developments, and these offer potential to enhance food systems. Some examples of where technology might contribute include soilless farming, widely supported by technology monitoring aspects such as temperature, humidity, CO_2_ light and nutrients; the ‘internet of things’ for precision agriculture, including promoting optimal environments for growth; blockchain technology for food traceability and safety; and e‐commerce platforms to support the food to farm concept (FAO, UNICEF, WFP and WHO, 2023). Together these technologies have the potential to reduce the miles that food travels, reduce food wastage, bring local producers in touch with markets, improve food safety, and improve information in the food system. A key element of the successful application of all this technology in the region will also be providing training in the technology for farmers and local workers within the food system.

There is a need to ensure nutritious diets for individuals living in poor urban environments within complex urban environments in an environmentally sensitive way to avoid exacerbating climate change effects on already fragile food systems (Pérez‐Escamilla & Moran, 2023). Investing in nutrition can provide benefits for poverty reduction and economic growth. For cities, such initiatives might include carefully designing and supporting urban and peri‐urban agriculture to promote and support the increasing demand for nutritious foods in urban areas using what others have termed ‘green’ approaches (Pérez‐Escamilla & Moran, 2023) to protect environments and to limit impacts on climate change. Similarly, initiating agricultural programmes such as direct farm‐consumer markets could improve agrifood systems and food security if provided with sustained support, along with country‐wide double‐duty actions, such as scaling up access to healthcare, redesigning cash and food transfers, subsidies and vouchers, and devising new nutritional guidelines for food in and around educational institutions. Small‐to‐medium enterprises constitute at least half of the food system and are fundamental to transform the ways food is produced and consumed. Targeted support to those actors is essential to reduce the cost of nutritious food and thereby increase affordability and enhance resilience of the overall urban food system. The impact of COVID‐19 has reinforced the need for food systems in the Asia‐Pacific region to become more nourishing, sustainable, equitable and resilient.

## CONFLICT OF INTEREST STATEMENT

The authors declare no conflict of interest.
